# Risk-based Algorithm-guided Treatment Protocol for the Management of Neovascular Age-related Macular Degeneration

**DOI:** 10.4274/tjo.galenos.2019.26235

**Published:** 2019-10-24

**Authors:** Murat Karaçorlu, Mümin Hocaoğlu, Serra Arf, M. Giray Ersöz, Işıl Sayman Muslubaş

**Affiliations:** 1 İstanbul Retina Institute, İstanbul, Turkey

**Keywords:** Anti-vascular endothelial growth factor, individualized medicine, neovascular age-related macular degeneration, treat and extend dosing

## Abstract

**Objectives:**

To assess outcomes of a risk-based algorithm-guided treatment protocol for neovascular age-related macular degeneration.

**Materials and Methods:**

Two hundred and ten eyes of 184 patients managed with anti-vascular endothelial growth factor (anti-VEGF) agents according to a protocol consisting of one of three initial regimens depending on risk with at least 2 years of follow-up were retrospectively evaluated. The “short-term monthly injections” protocol was used for low-risk patients with low-risk lesions and good fellow-eye vision. Patients with low-risk lesions but without good fellow-eye vision, or those with good fellow-eye vision and high-risk lesions were managed according to the “short-term treat-and-extend (TREX)” protocol. The “extended TREX” protocol was for patients with high-risk lesions and low fellow-eye visual acuity.

**Results:**

The initial treatment plan consisted of short-term monthly injections in 62 eyes (30%), the short-term TREX regimen in 120 eyes (57%), and the extended TREX regimen in 28 eyes (13%). Overall, 63% of cases met the criteria for cessation of treatment. Approximately 58% of these cases had recurrence, at a mean of 13 months. The mean change in VA from baseline was +9.0 letters at 12 months and +8.0 letters at 24 months. VA improved during a mean follow-up of 46.8±22 months, with a mean of 3.4±1.6 anti-VEGF injections per year.

**Conclusion:**

The risk-based algorithm-guided treatment protocol yielded visual outcomes similar to those of the common alternative treatment and monitoring regimens, with a dramatically reduced number of injections, as required by the individual lesion and vision in the fellow eye.

## Introduction

Age-related macular degeneration (AMD) is the leading cause of vision loss and blindness among people aged 50 years and older in industrialized countries. Neovascular AMD (nAMD) affects only 10-15% of AMD cases, but accounts for more than 80-90% of cases of severe visual impairment.^1,2^ The efficacy and safety of intravitreal anti-VEGF treatment (bevacizumab, ranibizumab, and aflibercept) has been demonstrated in multiple clinical trials and remains the initial treatment option for nAMD.^3,4,5,6,7,8,9,10^

Neovascular AMD includes a broad spectrum of genetic backgrounds and associated phenotypes. Unfortunately, individual responses to anti-VEGF treatment show substantial heterogeneity, and most eyes exhibit recurrent or resistant exudative features. Appropriate dosing of anti-VEGF therapy for patients with nAMD is essential for achieving the desired therapeutic outcomes. A fixed dosing regimen (monthly or bimonthly) has considerable visual acuity (VA) benefit.^3,4,7,13^ However, frequent treatments are excessive for most patients and cause an economic burden and increase the risk of ocular and systemic side effects.^13^ For this reason an individualized as-needed (PRN; pro re nata) dosing regimen involving close individualized monitoring and reactive treatment upon signs of disease activity has been widely adopted in clinical practice. Although the PRN therapy can reduce the number of injections, monthly assessment visits are still required to detect disease recurrence promptly. This places a heavy burden on clinicians and patients. At the same time, large-scale prospective trials and real-life studies have shown that these regimens often yield inferior visual outcomes, probably because of undertreatment, as shown by the low mean number of visits and injections.^5,6,15,16^ The treat-and-extend (TREX) regimen, which attempts to take a proactive approach and tailor the treatment to the response of an individual patient, is becoming increasingly popular. This treatment regimen is associated with significantly fewer patient visits, injections, and annual direct medical costs than monthly injections, as shown in phase III trials.^10,11,12^ Potential criticisms of the TREX approach include the possibility of overtreating a dry retina, an increased risk of atrophy, greater cost, and the need for treatment discontinuation criteria.

Neovascular AMD is a complex and chronic disorder. It is obvious that current treatment strategies may not be cost-effective, as the expected costs for a patient with newly diagnosed nAMD may reach $250,000 over 20 years.^17^ A treatment strategy consisting of possibly indefinite anti-VEGF injections poses a financial, but also a social and psychological burden on elderly patients with other systemic comorbidities. It is known that a significant number of patients delay or discontinue treatment, and the early benefit gained from treatment could be lost over time. In observational studies, the number of patients who are lost to follow-up ranged between 17% and 34% at 1 year, between 16% and 47% at 2 years, to approximately 50% at 4-5 years.^18^ Now the aim of therapy is shifting from merely saving distance VA to maintaining a good quality of life, reflecting the influence of treatment on daily living activities and emotional wellbeing.^18^

Treatment intervals and the number of injections need reassessment. Extensive research efforts have been directed to determining optimal management strategies for nAMD. A suitable treatment regimen remains an aim for individualized medicine.^19^

In this study, we describe a simple guide to risk classification according to lesion morphology and VA in the fellow eye, which is adjusted to real-life requirements. Also, we propose individualized therapeutic and treatment discontinuation criteria for patients treated with anti-VEGF agents for nAMD. We define this approach as a risk-based algorithm-guided treatment protocol. Rates of choroidal neovascularization (CNV) recurrence, the number of injections, and the VA outcomes using the proposed treatment approach have been evaluated.

## Materials and Methods

This study was a retrospective chart review of patients with a diagnosis of nAMD who were managed with the newly defined “Risk-based Algorithm-guided Treatment Protocol” in a retina-only practice clinic (İstanbul Retina Institute, İstanbul, Turkey). The study protocol was approved by the ethics committee of Şisli Memorial Hospital, Istanbul. The study was performed in accordance with the tenets of the Declaration of Helsinki. Written informed consent was obtained for each patient before anti-VEGF intravitreal therapy.

### İstanbul Retina Institute’s Protocol for Neovascular Age-related Macular Degeneration

The clinical risk assessment and stratification were based on the morphological features of CNV and the VA in the fellow eye (Table 1). According to our stratification of the lesions, larger classic and occult CNV lesions (>1 disc area), polypoidal choroidal vasculopathy (PCV), and retinal angiomatous proliferation (RAP) lesions require substantial attention and are considered high-risk. VA of less than 20/63 in a newly diagnosed nAMD patient suggests the need for careful monitoring and appropriate treatment. From our experience, this is an important risk factor for visual impairment. As a result, patients were classified into three risk groups.

Treatment strategies and regimens according to risk are presented in Table 2.

1) The short-term monthly injection protocol is used in low-risk patients with low-risk lesions and vision in the other eye that is adequate for everyday social activities. The treatment protocol consists of three intravitreal injections of anti-VEGF at monthly intervals (30±7 days) until the disease is inactive. From injection 3 and upon a dry macula on optical coherence tomography (OCT), patients undergo follow-up, initially monthly, and then, if the macula looks dry, with stepwise 2-week interval increase, to a maximum of a 3-month interval, until signs and symptoms of recurrent exudative activity are detected. Upon early recurrence (within 12 months after treatment cessation), the short-term TREX regimen is initiated. Upon late recurrence (12 months after treatment cessation), short-term monthly injections are re-initiated.

2) Patients with low-risk lesions but without good fellow-eye vision or those with good fellow-eye vision and high-risk lesions are classified as intermediate-risk patients and are managed according to the short-term TREX protocol. The short-term TREX protocol consists of a minimum of three monthly injections, until a dry macula is observed on OCT. Visit and treatment intervals are extended by 2 weeks. If there is increasing fluid on OCT, then the intervals are reduced by 2 weeks. The short-term TREX protocol is continued until treatments have been extended to a 3-month interval and patients have received at least eight intravitreal injections. After injection 8, if the macula is dry at the third 3-monthly visit, the treatment is stopped. Patients continue to be evaluated at 3-month intervals. Upon early recurrence, the extended TREX regimen is initiated. Upon late recurrence, the short-term TREX regimen is re-initiated.

3) The extended TREX protocol is for high-risk patients with high-risk lesions and low fellow-eye VA (i.e., those with a high risk of progression to bilateral blindness). The extended TREX protocol is initiated and implemented following the algorithm described above. In the 36 months after protocol implementation, if the macula is dry at each of three consecutive 3-monthly visits, then stopping treatment is considered. After treatment has been stopped, patients are followed up at 3-month intervals for any signs of recurrence. Upon recurrence at any follow-up time, the extended TREX regimen is re-initiated. If at any point during the treatment schedule patients fail to respond (no decrease in fluid or increase in VA) or if treatment response is inadequate (increasing fluid, decreasing vision, or both, related to the CNV process) as determined by VA and OCT findings, the anti-VEGF agent is switched to another agent or, in cases of PCV, a combination of photodynamic and anti-VEGF therapy.

### Data Collection

Medical records of 385 consecutive patients managed with anti-VEGF therapy for new nAMD between January 2010 and June 2018 were reviewed. Patients with irregular follow-up examinations and treatments, and those having less than 24 months of follow-up were excluded.

Exclusion criteria were: prior treatment of CNV in the study eye, advanced lesions composed of subfoveal and juxtafoveal fibrosis, geographic atrophy, retinal pigment epithelial tears, and extensive submacular hemorrhage.

All patients had been diagnosed with nAMD on the basis of clinical characteristics and multimodal imaging including spectral domain-OCT (Spectralis, Heidelberg Engineering, Heidelberg, Germany), fluorescein angiography (FA), and indocyanine green angiography (ICGA) (particularly in cases of suspected PCV and RAP) and treated by two experienced retinal specialists (M.K. and S.A.) at a single institution. Indications for anti-VEGF therapy included hemorrhage and/or lipid exudation on ophthalmoscopy, presence of intraretinal and/or subretinal fluid accumulation with or without hyperreflectivity suggestive of CNV on OCT scan or any evidence of CNV disease activity on FA or ICGA. Treatments initially included intravitreal injections of bevacizumab (1.25 mg), ranibizumab (0.5 mg), or aflibercept (2.0 mg).

At every visit, patients were evaluated with OCT and best corrected VA was assessed by using ETDRS charts. FA and/or ICGA were performed at initial presentation and at other times at the discretion of the investigator. Patients were advised to return to the clinic sooner than scheduled if they noted any visual disturbance. If at any time there was a recurrence, as determined by clinical examination and OCT, treatment was re-initiated immediately.

Re-treatment criteria after discontinuation of therapy were: vision loss of ≥5 letters, intraretinal or subretinal fluid on OCT, or new hemorrhage. Extension criteria were based on absence of the following: macular fluid on OCT, vision loss of ≥5 letters, new macular hemorrhage, and increased lesion size or leakage on FA or ICGA.

### Statistical Analysis

Pearson’s chi-square tests were used to compare categorical variables. Student’s t-test was used to explore differences in means among continuous variables. One-way analysis of variance (ANOVA) was used to compare means of three or more independent groups. Tamhane’s test was used for post-hoc comparison of baseline VAs between initial treatment groups. A repeated-measures ANOVA was used to compares means across three or more repeated measures of VA. The Bonferroni post-hoc test was used to compare VA after cessation of anti-VEGF therapy and recovery of vision after the treatment re-institution due to recurrence of CNV. A p value less than 0.05 was considered statistically significant.

## Results

Among 385 patients, 184 (210 eyes) met the inclusion criteria for the study cohort. The baseline demographic and clinical characteristics of participants in each initial treatment plan are detailed in Table 3. There were no significant differences in age and sex between the three initial treatment groups. About 14% (26/184) of the participants had bilateral study-eligible nAMD. No significant difference in lesion characteristics was observed between the short-term TREX and extended TREX groups. Mean baseline VA in the short-term TREX group was worse than in the short-term monthly injection group (p=0.003).

**Figure 1:**
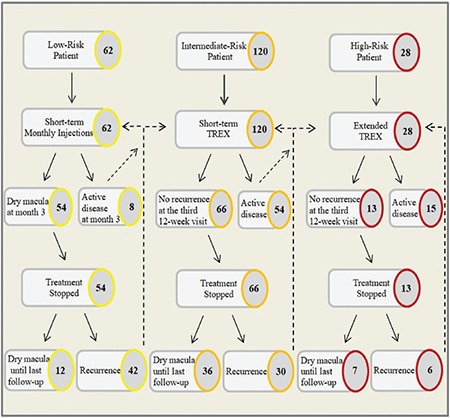
A flowchart of the study cohort showing the distribution of patients and step-by-step directions for the proposed risk-based algorithm-guided treatment protocol

**Figure 2:**
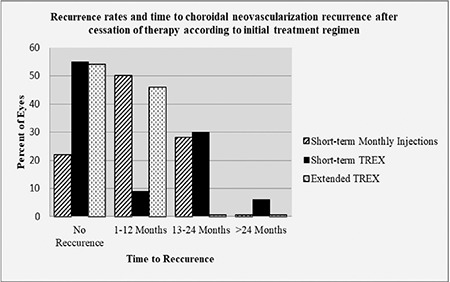
Recurrence rates and time to choroidal neovascularization recurrence after cessation of therapy. **Initial treatment approach short-term monthly injections:** Overall, 87% (54/62) of the eyes met the necessary requirements for ceasing therapy. About 22% (12/54) of the eyes showed no recurrence during mean follow-up 47 months (range, 22–71 months), while 50% (27/54) of the eyes showed recurrence at 2–12 months, and 28% (15/54) showed recurrence at 13–24 months after cessation of therapy. **Initial treatment approach short-term TREX regimen:** Overall, 55% (66/120) of the eyes met the necessary requirements for cessation of therapy. About 55% (36/66) of the eyes showed no recurrence during mean follow-up 25 months (range, 9–66 months), while 9% (6/66) of the eyes showed recurrence at 2–12 months, 30% (20/66) showed recurrence at 13–24 months, and 6% (4/66) showed recurrence at >24 months after cessation of therapy. **Initial treatment approach extended TREX regimen:** Overall, 46% (13/28) of the eyes met the necessary requirements for cessation of therapy after mean 17 injections (range, 15–23). About 54% (7/13) of the eyes showed no recurrence during mean follow-up 15 months (range, 9–24 months), the remaining 46% (6/13) showed recurrence at 4–12 months (mean, 6.5 months) after cessation of therapy.

Overall, 133 eyes (63%) completed the initial planned treatment regimen and met the criteria for cessation of therapy. The remaining 77 eyes did not meet the criteria, and treatment was resumed in a stepwise manner, as determined by the protocol. Of the eyes that completed the initial planned treatment regimen and for which treatment was stopped, 78 (59%) showed recurrence, and additional treatment was needed. A flowchart of the study showing the distribution and step-by-step directions regarding the algorithm is presented in Figure 1. The overall average time from completion of the initial treatment regimen to recurrence of CNV was 13.0±10.2 months (range, 2-43 months). The mean intervals from discontinuation of treatment to recurrence in the short-term monthly injection, short-term TREX, and extended TREX groups were 10.8±10 months (range, 2-24), 17.8±8 months (range, 2-43), and 6.2±2 months (range, 4-12), respectively. The recurrence interval for the short-term TREX group was significantly longer than for the short-term monthly injection (p=0.008) and extended TREX groups (p<0.001). Details are presented in Figure 2.

Mean VA after initial treatment with the short-term TREX regimen was significantly lower than the short-term monthly injection regimen at 12 months (20/38 vs 20/30) and 24 months (20/40 vs 20/30) (p=0.009 and p=0.001, respectively). The percentage of eyes with VA ≥20/40 at 12 months and 24 months was 57% and 52%, respectively. The proportions of patients at 12 months who had VA 20/40 or better after initial short-term monthly injections, short-term TREX, and extended TREX regimens were 82%, 64%, and 71%, respectively, slightly higher than for those who had VA 20/40 or better at 24 months (77%, 60%, and 57%, respectively). There was no patient with Snellen equivalent VA 20/200 or worse at 12 or 24 months.

Overall VA had improved significantly after 12 and 24 months of treatment (p<0.001). However, VA decreased in the subsequent years of treatment, but remained higher than baseline at 60 months after treatment. Overall, 66 eyes (31%) and 68 eyes (32%) gained ≥15 ETDRS letters and 4 (1.9%) and 10 (4.7%) eyes lost ≥15 letters from baseline to 12 months and 24 months, respectively. VA improved from 63.5 letters at baseline to 72.5 (+9.0) and 71.5 (+8.0) letters at 12 and 24 months, respectively. The overall mean VA at last follow-up was 20/47 (range, 20/20-20/400). There was no difference in mean VA at last visit between the three initial treatment groups. The overall mean follow-up period was 46.8±22 months (range, 24-92 months). There was no difference between the three initial treatment groups in mean follow-up duration.

The mean number of injections after initial short-term monthly injections (n=4.7) was significantly lower than number of injections after the initial short-term TREX (n=7.4) and extended TREX (n=7.8) regimens at 12 months (p<0.001). There were significant differences in the number of injections between the initial short-term monthly injection (n=8.5), short-term TREX (n=10.7), and extended TREX (n=13.2) regimens at 24 months (p<0.001). No significant differences in the number of injections between the initial short-term monthly injection (n=14.8) and short-term TREX (n=16.2) regimens was observed at the last visit, while the extended TREX (n=23.2) regimen group had a higher number of injections than these groups (p=0.001). Patients received a mean of 17.0±10 (range, 3–56) injections over a mean follow-up period of 46.8±22 months. The mean number of injections per year was 3.4±1.6 (range, 3-13).

The overall mean yearly rate of change in VA and number of injections from baseline are shown in Table 4. Comparison of visual outcomes and number of injections between recent landmark clinical trials and our study population is presented in Table 5.

VA after CNV recurrence was compared with VA before the recurrence. The overall mean best corrected VA before initial recurrence was 20/38 and decreased significantly to 20/51 after recurrence (p<0.001). The mean VA of 20/44 in the period after re-institution of therapy was significantly lower than the mean VA before recurrence of CNV (p=0.001). There was no significant difference between the short-term monthly injection and extended TREX groups in mean VA before the recurrence and in the period after re-institution of therapy. Consequentially, the mean best corrected VA of 20/51 obtained in the period after re-institution of therapy was significantly lower than the mean VA of 20/44 before recurrence of CNV in patients managed with the short-term TREX regimen (p=0.01).

About 87% (n=54) of eyes in the short-term monthly injection group were fairly dry after three injections. Nineteen of these patients who completed the protocol and subsequently had recurrence were able to complete additional round(s) of short-term monthly injections (n=8), short-term TREX (n=10), and one patient completed a combination of short-term TREX and extended TREX regimens. Two patients from the short-term monthly injection group who did not meet the criteria for discontinuation of therapy were able to complete round(s) of short-term TREX and one completed an extended TREX regimen. About 55% (n=66) of eyes in the short-term TREX group were dry after eight injections. Fourteen patients who completed the short-term TREX protocol and subsequently had recurrence were able to complete additional round(s) of the short-term TREX protocol and three were able to complete the extended TREX protocol. Four patients who did not meet the criteria for discontinuation after initial short-term TREX were able to complete round(s) of the extended TREX regimen. Two patients who completed the extended TREX protocol and subsequently had recurrence were able to complete one additional round of the extended TREX protocol.

Most eyes received bevacizumab (65%, 136/210), aflibercept (11%, 23/210), or ranibizumab (2%, 4/210) treatment. The remaining eyes received a combination of bevacizumab and aflibercept (17%, 36/210), ranibizumab and aflibercept (2%, 4/210), or all three (3%, 7/210).

At baseline, 36% (75/210) of the eyes were pseudophakic. During follow-up, 13% (28/210) of the eyes underwent cataract surgery. About 21% (45/210) of the eyes had some degree of cataract at the last visit.

The proportion of eyes not lost to follow-up, before data collection, was 66% (139/210). The causes of loss to follow-up in the remaining 71 eyes included: death (n=19), relocation, missed or delayed examination due to systemic disease, or an unknown reason (n=52).

## Discussion

This study investigated a cohort of treatment-naïve nAMD patients treated with anti-VEGF agents using a newly defined risk-based algorithm-guided treatment protocol based on individualized stratification according to the risk of visual impairment. This single-center retrospective series was managed by two physicians (M.K. and S.A.) over a period of 8.5 years. The VA outcomes obtained at 1 and 2 years were comparable to those in the large randomized trials of anti-VEGF therapy for nAMD and were maintained long term with continued treatment after a mean follow-up of 47 months. These benefits suggest that sustained long-term visual gains can be achieved in real-world settings with a significantly reduced number of anti-VEGF treatments (an average 3.4 injections per year), reducing loss to follow-up in the management of nAMD with a risk-based algorithm-guided treatment protocol.

Visual impairment following inappropriate management of nAMD has serious negative effects on patients’ independence, productivity, and quality of life. Dilated fundus examination and use of advanced imaging modalities are essential for nAMD diagnosis and monitoring. Although there is no cure, timely and continuous treatment with intravitreal anti-VEGF injections is improving or maintaining VA and, on the basis of clinical trials, forms the mainstay of treatment.^3,4,5,6,7^ On the other hand, it is well known that real-world challenges and unmet needs pose significant barriers to treatment goals, and unfortunately, visual outcomes in real-world evidence studies are usually worse.^18^

Frequent physician visits and imaging as well as therapy consisting of an uncertain number of anti-VEGF injections cause a significant burden on both patient and medical staff. Intravitreal injections may be associated with serious ophthalmic and systemic adverse events. Suboptimal outcomes can be associated with many complex factors: a significant number of patients delay or discontinue treatment owing to poor response, progression of untreatable aspects of the disease, or for financial and social reasons. Many questions relate to the optimal treatment regimen and duration, the frequency of follow-up and re-treatment, and which patients can discontinue treatment.^20^

Today, fixed dosing, PRN, and TREX regimens are offering the opportunity for a better balance of practicality and effectiveness when selecting the most appropriate treatment regimen. Because the effect of anti-VEGF agents is related to many complex factors, the benefit of therapy varies among patients. Consequently, optimal results cannot be obtained with any single regimen. This has encouraged us to develop a strategic plan for improving patient adherence to therapy and long-term visual benefit while optimizing follow-up and injection frequency. Our risk-based management strategy is based on recent scientific evidence and provides a risk classification according to CNV lesion morphology and VA in the fellow eye.

According to scientific evidence, some types of lesion are commonly associated with short- and long-term VA loss.^21,22,23,24^ Post-hoc analyses of major phase III trials showed that eyes with the smallest lesions (≤1 disc area) had VA gains of approximately 10 ETDRS letters more than patients with the largest lesions.^21,22^ Additionally, a larger baseline CNV area has been associated with a higher risk of fibrotic scar formation.^#*#ref23#*^# Larger baseline CNV lesions and the presence of baseline retinal pigment epithelium (RPE) elevation remain independently associated with worse short- and long-term VA.^24^ Evidence from real-life studies has also shown significantly negative correlation between lesion area and visual change, and it has been suggested that individualizing anti-VEGF therapy on the basis of initial lesion characteristics could be a valuable approach.^25^ It seems clear that baseline angiographic characteristics, such as larger CNV lesions, and OCT characteristics, such as greater subretinal tissue complex thickness, at baseline predict increased risk of VA loss. We strongly believe that larger CNV lesions deserve more attention, and according to our morphological classification criteria, are determined as lesions with high risk.

PCV is believed to be a subtype of nAMD.^26^ The role of VEGF in the pathogenesis of PCV is not fully understood, and the optimal treatment strategy remains unclear. Based on clinical trial data, anti-VEGF monotherapy performed by PRN or fixed dosing can achieve anatomical and functional improvement and could be considered as first-line treatment for PCV.^27,28^ There are limited data on the management of PCV with a TREX regimen using anti-VEGF agents. Recently, Pak et al.^29^ reported outcomes of a TREX regimen using ranibizumab to treat 29 PCV patients for 12 months. The mean number of injections was 7, and after the loading phase, 41% (12/29) of the eyes had no recurrence.

It has been proposed that the number of injections could be expected to be higher when performed as monotherapy than when performed in combination with photodynamic therapy. Patients who were not receiving multiple injections (average, 7-8) over 12 months could not achieve the functional outcomes reported in clinical trials.^26,27^ In view of the evidence, PCV lesions show a broad spectrum of clinical characteristics and according to our risk determination deserve considerable attention.

RAP is recognized as a variant of nAMD, characterized by abnormal communication between the choroidal and retinal circulation.^30^ It has been proposed that as the anastomoses between the retinal and the choroidal circulation become more established, they become more resistant to anti-VEGF therapy.^31^ Early in their evolution, RAP lesions are generally accompanied by intraretinal changes exquisitely sensitive to intravitreal anti-VEGF agents, so early aggressive therapy is essential for preventing irreversible neurosensory damage.^32,33^ On the other hand, in RAP, choroidal thinning during continuous treatment may worsen RPE atrophy.^34^ Certain clinical features related to RAP lesions should be taken into consideration, and according to our risk estimation, these lesions are determined high risk.

Clinical evidence has demonstrated that the severity of AMD in one eye tracks disease severity in the fellow eye.^35^ This knowledge emphasizes the symmetrical nature of the disorder and has allowed us to be more certain when discussing prognosis, treatment, and monitoring strategies. It is well known that decreased VA is negatively associated with quality of life, and VAs between 20/50 and 20/100 cause decrements that require considerable help with daily functions.^36^ VA in the better eye of less than 20/63 is defined as low vision and less than 20/400 is defined as blindness. Additionally, VA less than 20/63 in the worse-seeing eye is defined as unilateral low vision and less than 20/400 is defined as unilateral blindness.^37^ Consequently, accelerated progression and inappropriate management of nAMD in a patient with low VA in their fellow eye could lead to restrictions in complex and social everyday activities. According to our criteria, fellow-eye VA of less than 20/63 in a patient with newly diagnosed nAMD is regarded as a significant risk factor for visual impairment and is considered a distinct entity establishing the protocol.

Multiple studies of monthly patient visits with PRN re-treatments have demonstrated that the number of injections varies between 3 and 24 over 2 years. The SUSTAIN study confirmed that approximately 20% of patients did not require re-treatment after the three initial monthly injections during the first 12 months, and 33% needed only one or two additional injections.^38^ This supports individualized dosing and further suggests that good responders may be overtreated with monthly or TREX dosing strategies. Identifying this limited patient population of good responders means that overtreatment could be minimized. Some small lesions may require only the loading dosing, and may not need any treatment during the following years. Our risk-based approach aims to isolate this limited patient number among groups we have described as low risk. Patients who require infrequent treatment (>12-month recurrence-free) could continue short-term monthly injections, but if disease recurs within a few months, the TREX regimen seems to be a more suitable approach. Consistent with previous studies, a significant proportion (up to 22%) of low-risk cases in our study cohort had no recurrence during a mean follow-up of 47 months (range, 22-71) after the treatment was stopped following three monthly injections. More importantly, about 50% of the eyes showed early recurrence within 12 months after treatment cessation; however, a significant number of these eyes were able to complete subsequent round(s) of strict short-term TREX or extended TREX regimens.

Little is known about the outcomes of patients who discontinue anti-VEGF therapy. In the CATT 5 study, about 15% of the patients received no treatments between the end of the trial and the follow-up study visit.^39^ Additionally, between the HORIZON exit and the SEVEN-UP evaluation, a mean of 3.4 years, 41% of study eyes received no treatment.^40^ Recently, outcomes of a new treatment strategy, described as a treat-extend-stop protocol, have been reported. As soon as patients with nAMD managed with a TREX protocol achieved anatomical stability, the therapy was stopped. Approximately 40% of the eyes were able to stop treatment after mean of 22 injections (range, 7-48). Approximately 70% of these eyes remained stable, and the remaining 30% showed recurrence during a mean follow-up of 14 months.^41^

In our study, 63% of cases met the criteria and had treatment discontinued after a mean 6.8 injections (range, 3-23). Approximately 58% of these eyes showed recurrence a mean 13 months (range, 2-33) after cessation of treatment. Interestingly, 55% of the eyes managed with short-term TREX were able to stop treatment after eight injections. This percentage is higher than the percentage of those who stopped treatment in the treat-extend-stop protocol and was achieved with a lower number of injections. In the Aflibercept Treat and Extend Therapy for Neovascular Age-Related Macular Degeneration (ATLAS) study, 12-week or longer treatment intervals were achieved in 35% of the patients during the first year and in 41% during the second year.^12^ The intervals for 68% of the patients in the TREX AMD Study were extended at the earliest possible visit or within one additional visit, and 30% had a macula that remained dry at every visit.^10^ All of the data suggest that a significant number of patients managed with the TREX protocol could achieve substantial anatomic stability with early extension. It is important to emphasize that the mean initial baseline VA in our study cohort was considerably better than in other studies. It is well known that a higher initial VA, smaller CNV lesion, and early diagnosis and treatment with anti-VEGF agents is associated with better outcomes.^25,43,44,45^ Consequently, the higher percentage in our study who achieved anatomical stability and could stop treatment after short-term TREX regimen could be explained by milder disease activity and prompt intervention. An important point that should be emphasized is that many patients treated with the treat-extend-stop protocol could stop therapy successfully and maintain improved vision even if the CNV recurred.^41^ Interestingly, in a significant number of patients who stopped treatment after short-term TREX, the vision loss after recurrence did not improve to the level of vision before recurrence. The recurrence of CNV after initial treatment cessation was associated with substantial mean VA loss of four letters. It is obvious that this last finding deserves special discussion and should be taken into consideration when discontinuation of scheduled treatment is planned.

In our study, the maximum mean gain in VA from baseline was recorded at 12 months and was largely maintained in more than 90% of the patients over a period of 36 months. It is noteworthy that the visual benefits obtained at 12 and 24 months were maintained to some extent long term in the subgroup of patients followed for 4 (n=94) and 5 years (n=60). However, mean VA at 6 years declined to 2.5 EDTRS letters worse than at baseline and 10.5 letters worse than at 2 years. There is limited evidence available on long-term follow-up in patients treated with anti-VEGF agents for nAMD. In the Comparison of Age-related Macular Degeneration Treatments Trials (CATT) follow-up study (mean 5.5 years), mean VA declined to 3.3 letters worse than at baseline and 10.8 letters worse than at 2 years.^39^ In the SEVEN-UP long-term follow-up study (mean 7.3 years), after baseline at entry into the ANCHOR or MARINA trials, there was a mean loss of 8.6 letters. From the therapeutic peak upon completion of 24 monthly injections in the ANCHOR or MARINA trials, mean vision had declined by 19.8 letters.^40^ However, when interpreting such results it is important to consider that these long-term trials also had significant loss to follow-up and this may have influenced visual outcomes. In addition, treatment effectiveness under routine clinical practice conditions differs from that in well-conducted controlled prospective clinical trials. More recently, some single-center retrospective studies have evaluated the efficacy of the TREX dosing regimen of anti-VEGF treatment in real-life conditions.^25,45^ Mrejen et al.^25^ presented results of 210 eyes that were managed with the TREX protocol for a mean of 3.5 years (range, 1-6.6 years). Maximal visual benefits from baseline were obtained at 18 months, and despite a slight decrease long term, were maintained for 3 to 6 years. Jaki Mekjavic and Zaletel Benda^45^ reported visual outcomes of 101 eyes that were continuously treated with anti-VEGF agents in the TREX protocol for 5 years. As a result, the improvement in VA was maintained for the first 3 years of treatment; however, after the fourth and fifth years of treatment, VA was not significantly different from baseline. Gillies et al.^46^ designed an observational study (Fight Retinal Blindness [FRB] Study) and analyzed the long-term outcomes of 1212 eyes treated with anti-VEGF agents for a mean 53.5 months. VA improved after 6 months and remained above the baseline VA for approximately 6 years. After 7 years, mean VA was 2.6 letters lower than baseline.

At 5 years, 55% (33/60) of our patients had VA ≥20/40, compared to the CATT follow-up study^39^ (50%), Gillies et al.^46^ (43%), and Jaki Mekjavic and Zaletel Benda^45^ (40%). Additionally, 20% (12/60) of our patients had VA ≤20/200, compared to the CATT follow-up study^39^ (20%), Gillies et al.^46^ (12%) and Jaki Mekjavic and Zaletel Benda^45^ (8%). However, it should be noted that there are differences between the studies, including the mean baseline VA (present study: 63.5 letters; CATT follow-up study^39^: 62.2 letters; Jaki Mekjavic and Zaletel Benda^45^: 60.5 letters; Gilles et al.^46^: 55.1 letters; Mrejen et al.^25^: 52 letters) and the mean age at first injection (present study: 74.0 years; CATT follow-up study^39^: 77.5 years; Jaki Mekjavic and Zaletel Benda^45^: 81.8 years; Gilles et al.^46^: 79.1 years; Mrejen et al.^25^: 81.1 years). It seems that our study had a younger cohort with better baseline VA. Mrejen et al.^25^ reported that older age at first injection correlated with worse VA in the short and long term. They also stressed that baseline VA and number of injections were predictors of visual change at all time points.^25^ As has been already shown in long-term studies, a substantial proportion of our patients experienced gradual vision loss over periods of 3 to 7 years from the initial benefits obtained at 2 years, which could be related to the irreversible progression of untreatable aspects of this complex condition (expansion of the size of the neovascular complex, scarring, atrophy, and persistence of fluid).^39,40^

The mean number of injections received by patients in our study population was 3.4 per year. The mean number of treatments (6.7) was highest in the first 12 months. However, the mean number of treatments gradually decreased during the subsequent 6 years of follow-up (3.7, 3.6, 2.9, 3.0, 1.9, and 1.8, respectively). In the CATT follow-up study, the mean number of treatments in the 3 years after the 2-year clinical trial protocol was higher (15.4) than in our study from years 3 to 5 (7.8).^39^ In the FRB study, the mean number of injections administered over the first year and over the second to seventh years were 6 and 5, respectively.^46^ Jaki Mekjavic and Zaletel Benda^45^ and Mrejen et al.^25^ reported 6.1 and 8.3 mean injections per year, respectively, with a continuous TREX approach. Real-world studies in nAMD treatment have found that patients receive fewer treatments than in clinical trials, which results in worse visual outcomes.^18^This could be associated, in part, with the treatment burden of frequent visits leading to decreased patient adherence. Interestingly, recent papers reporting long-term real-world outcomes using the TREX regimen have concluded that initial VA is more important in predicting VA after treatment than the number of intravitreal injections received,^45^ and patients with better initial VA preserve good VA after long-term treatment.^25^ There is no doubt that prompt diagnosis and treatment at onset of nAMD is therefore essential. On the other hand, individualization of therapy is a current trend. In order to individualize therapy, we initially estimated the risk of visual impairment (initial lesion composition and fellow-eye VA) and initiated a treatment strategy that plays a key role in determining the injection number and injection intervals. Patients in our study had fewer visits and treatments, owing to the nature of the treatment protocol, thus reducing the treatment burden. The proportion of patients lost to follow-up (34%) for the entire cohort was better than previously reported.^25^

Despite the success of anti-VEGF therapy in restoring vision and preventing damage associated with CNV, there has been increasing concern that anti-VEGF therapy may increase the risk of RPE atrophy in eyes with neovascular AMD. Some studies have identified an association between the number of anti-VEGF treatments over time and the growth rate of RPE atrophy.^34,47,48^ It is unclear whether anti-VEGF therapy accelerates or increases the risk of macular atrophy. However, while the relationship is unclear, the number of anti-VEGF interventions should be limited to the minimum required to control the disease.^32^ Because a higher treatment rate is associated with better VA results but could increased the risk of atrophy, an individualized therapeutic approach may keep the right balance between too many and too few treatments.^25^ To obtain long-term results, we have proposed a strategic plan based on initial lesion composition and risk of visual impairment, in which re-treatment and follow-up periods are adjusted according to patients’ responses to therapy. According to our data, it appears reasonable to consider discontinuing treatment when anatomical stability is achieved in order to minimize the burden of treatment and potential for atrophy. However, it should not be forgotten that patient adherence to follow-up plays a key role in reducing the risk of complications associated with recurrent CNV activity.

### Study Limitations

There are inherent limitations to our study that need to be carefully considered, including its retrospective nature and single-center design. Additionally, the therapeutic agents available during our long follow-up period (up to 92 months) have changed, and the number of patients at each extended follow-up period of 6 and 7 years is small. A significant proportion of patients had some degree of cataract, which could affect the VA outcomes. The study population included bilateral cases, in which both eyes should be treated simultaneously. The results of the study may be difficult to interpret and not easily comparable because the risk-based protocol represents a unique approach in the management and monitoring of nAMD. Additionally, this treatment approach could be much more complex than presented here.

## Conclusion

This study represents a treatment approach that takes into account real-life requirements and challenges in the management of nAMD on the basis of current evidence from clinical trials on anti-VEGF therapy. The risk-based algorithm-guided treatment protocol yielded visual outcomes similar to the common alternative treatment and monitoring regimens with a dramatically reduced number of injections as required by the individual patient pathology and vision in the fellow eye. The favorable functional and anatomical outcomes obtained in our study with a lower number of injections could be attributable to many factors: a younger study cohort, higher baseline VA (early presentation, mild CNV activity), prompt diagnosis, improved patient adherence, and strict regimentation of anti-VEGF injections and monitoring by the clinician. The risk-based algorithm-guided treatment protocol holds potential to provide clinicians and patients the opportunity for optimal vision gains and anatomic disease control with substantially decreased treatment burden and noncompliance, as well as a lower cumulative risk of injection-related adverse events. Additionally, preplanning of the injections enables optimization of use of the medical staff and technical resources. Despite some limitations, we believe our research findings are important in guiding routine clinical practice.

## Figures and Tables

**Table 1:**
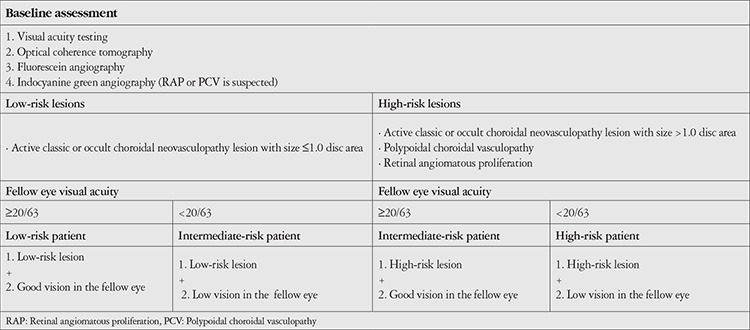
Risk-based algorithm approach: risk classification according to the morphological characteristics of the lesion and risk assessment according to visual acuity in the fellow eye (treatment-naïve eyes)

**Table 2:**
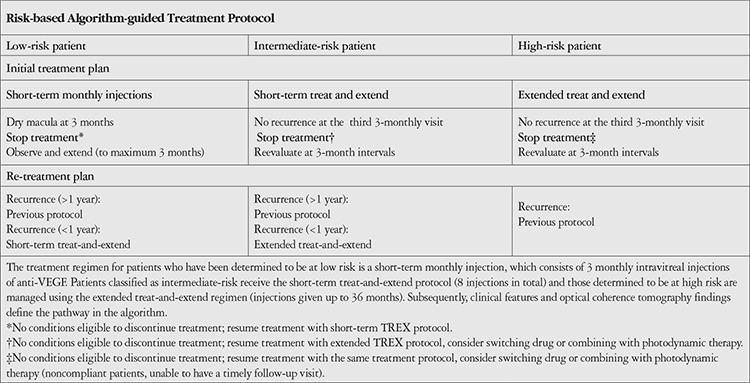
Flow chart for management of patients with neovascular age-related macular degeneration according to the risk-based algorithm approach

**Table 3:**
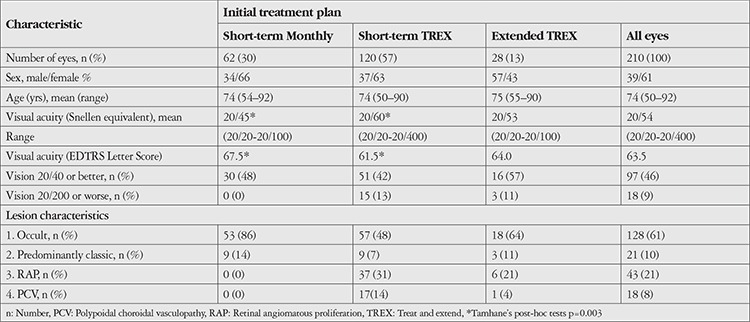
Baseline demographic and clinical characteristics of patients managed with risk-based algorithm-guided treatment protocol for neovascular age-related macular degeneration

**Table 4:**
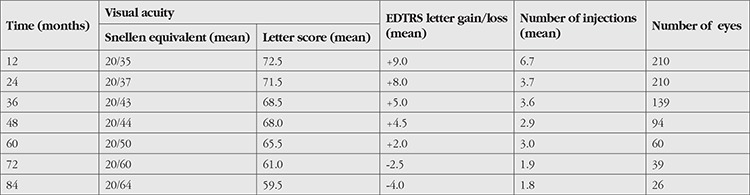
Overall mean yearly rate of change from baseline in visual acuity and number of injections

**Table 5:**
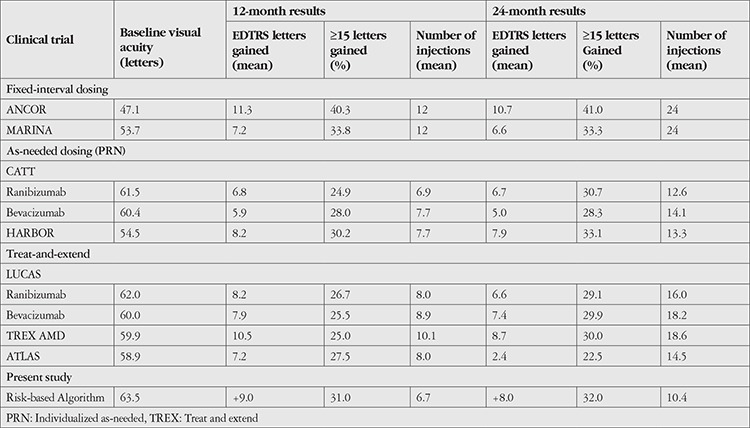
Findings of representative fixed dosing, as-needed, and treat-and-extend trials of anti-VEGF therapies compared with the risk-based algorithm-guided treatment protocol
